# Gene Expression Differences Between Young Adults Based on Trauma History and Post-traumatic Stress Disorder

**DOI:** 10.3389/fpsyt.2021.581093

**Published:** 2021-04-08

**Authors:** Kaitlin E. Bountress, Vladimir Vladimirov, Gowon McMichael, Z. Nathan Taylor, Gary Hardiman, Dongjun Chung, Zachary W. Adams, Carla Kmett Danielson, Ananda B. Amstadter

**Affiliations:** ^1^Virginia Institute for Psychiatry and Behavioral Genetics, Virginia Commonwealth University (VCU), Richmond, VA, United States; ^2^Department of Psychiatry and Behavioral Sciences, College of Medicine Texas A&M University, Richmond, VA, United States; ^3^Lieber Institute for Brain Development, Johns Hopkins University, Baltimore, MD, United States; ^4^Institute for Global Food Security, Queens University Belfast, Belfast, United Kingdom; ^5^Department of Public Health Sciences, Medical University of South Carolina, Charleston, SC, United States; ^6^Department of Psychiatry, Indiana University of Medicine, Indianapolis, IN, United States; ^7^National Crime Victim Research and Treatment Center, Medical University of South Carolina, Charleston, SC, United States; ^8^Department of Psychiatry and Behavioral Sciences, Medical University of South Carolina, Charleston, SC, United States

**Keywords:** gene expression, gene network analyses, trauma exposure, post-traumatic stress disorder, civilian trauma, veterans

## Abstract

**Background:** The purpose of this study was to identify gene expression differences associated with post-traumatic stress disorder (PTSD) and trauma exposure (TE) in a three-group study design comprised of those with and without trauma exposure and PTSD.

**Methods:** We conducted gene expression and gene network analyses in a sample (*n* = 45) composed of female subjects of European Ancestry (EA) with PTSD, TE without PTSD, and controls.

**Results:** We identified 283 genes differentially expressed between PTSD-TE groups. In an independent sample of Veterans (*n* = 78) a small minority of these genes were also differentially expressed. We identified 7 gene network modules significantly associated with PTSD and TE (Bonferroni corrected *p* ≤ 0.05), which at a false discovery rate (FDR) of *q* ≤ 0.2, were significantly enriched for biological pathways involved in focal adhesion, neuroactive ligand receptor interaction, and immune related processes among others.

**Conclusions:** This study uses gene network analyses to identify significant gene modules associated with PTSD, TE, and controls. On an individual gene level, we identified a large number of differentially expressed genes between PTSD-TE groups, a minority of which were also differentially expressed in the independent sample. We also demonstrate a lack of network module preservation between PTSD and TE, suggesting that the molecular signature of PTSD and trauma are likely independent of each other. Our results provide a basis for the identification of likely disease pathways and biomarkers involved in the etiology of PTSD.

## Introduction

Between 50 and 70% of individuals experience at least one trauma in their lifetimes (1). Of those individuals, 8–32% develop post-traumatic stress disorder [PTSD; (2, 3)]. PTSD is a serious disorder associated with medical ailments, suicide, and early mortality, but major gaps remain in our understanding of its etiology (4–6). PTSD is heritable, with estimates ranging between 24 and 72% (7–9). Large-scale genome wide association studies (GWAS) are revealing key influences on PTSD.

Recent GWAS of PTSD, PTSD total symptoms, and/or its symptom clusters (e.g., re-experiencing symptoms) (10–12) have revealed a few hits that differentiate PTSD cases and controls, and more when examining PTSD symptoms as a quantitative trait and/or when examining continuous symptoms within clusters. One of the most consistent findings is that genes implicated in cell cycle control and other mental health conditions such as schizophrenia and bipolar (*MAL1L1*) are associated with PTSD phenotypes. The effects found in both samples involve genes implicated in intracellular protein transport associated with risk-taking (*TSNARE1*) as well as nucleic acid binding which is important for cognitive abilities (*EXD3*). Additionally, genes important for steroid signaling, hormone metabolism, and stress response (CRHR1, HSD17B11), central nervous system development (TCF4), and transcriptional activity and enhancer functions (i.e., ZDHHC14, PARK2, chr13: 55,652,129–55,759,209; 11) were also found to be important.A review of PTSD GWAS summarizes key findings, with several significant SNPs differentiating cases and controls, coming from genes in systems important for regulating circadian rhythm, synaptic processes, immune function, and neuroplasticity [e.g., LINC01090, BC036345, ZNRD1-AS1, RORA, NLGN1, TLL1; (13)]. Despite these advances, questions remain about how genetic factors relate to PTSD and whether observed patterns are specific to PTSD or are related to trauma exposure broadly. Because PTSD is more prevalent in women than men and genetic influences on PTSD are stronger among women than men (7, 14), studies are needed to characterize the genetic correlates of PTSD and trauma exposure (TE) in women.

### Gene Expression Studies

Studying gene expression is essential for our understanding of the PTSD pathophysiology (15, 16). While trauma exposure precedes all cases of PTSD, not all people with TE will develop PTSD. Therefore, expression studies are needed that include participants with or without TE and with or without PTSD to disentangle whether observed genetic differences are influenced by TE or PTSD. Previous post-mortem expression studies have reported expression differences between subjects with PTSD and controls in the dorsolateral pre-frontal cortex, which regulates fear-based responses (17). Research examining the post-mortem brains of people who died of suicide with and without child abuse suggests differences in the expression of genes important for myelination (18). A study of transcriptome-wide analyses of gene expression changes in post-mortem brains of individuals with PTSD identified genes involved in synapse, neuron and axon terms, as well as glia formation, actin binding, and small GTPase signaling (19). This same study found that the global transcriptomic signatures for PTSD closely resemble schizophrenia, autism, and bipolar disorder, consistent with GWAS of PTSD (14, 20). Thus, there is at least some convergence in the pathology of PTSD using blood and brain tissues. Indeed, as the collection of post-mortem brain tissue involves ethical, logistic, and technical confounds (21, 22), blood is increasingly viewed as a viable and valid proxy tissue for gene expression data. Furthermore, several studies have pointed to a reasonable correlation of the total gene expression between different tissue, notably brain and blood (23–25). Finally, studying blood related gene expression is also advantageous as some processes underlying PTSD are reflected in peripheral blood gene expression [e.g., glucocorticoid sensitivity, (26)]. For all of these reasons, gene expression data extracted from whole blood is becoming increasingly commonplace.

Comparing gene expression profiles between subjects with PTSD and TE have suggested genes involved in the hypothalamic-pituitary-adrenal Axis (HPA) [e.g., *FKBP5*; (27–30)] and signal transduction processes [e.g., *STAT5B*; (16, 28)]. Most studies included individuals in the TE control group if individuals did not meet criteria for *current* PTSD, which is problematic, since differences may be obscured between those who previously were diagnosed with PTSD vs. those never diagnosed. A study of subjects with PTSD to those without TE found evidence for gene expression differences that are important for activating the adaptive cellular immune response (e.g., *IL-12* and *IL-18*) (31), but lack of a non-PTSD TE group limits conclusions about molecular genetic risk.

#### Gene Network Analysis

A limitation of studies utilizing single gene expression analyses is the focus on ranking a list of individual differentially expressed genes with the highest statistical significance. This selection approach is somewhat arbitrary, does not consider the potential interaction between genes in the dataset, and does not provide a broader, system-level view of expression. Thus, a network approach can be used to better understand the functional changes between the disease and normal transcriptomes. One study (32) using this approach identified over-expression of genes enriched for innate-immune response and interferon signaling (PTSD-TE), while another (33) found differences for genes enriched for signal transduction (PTSD-control).

### Current Study

The few previously reported gene expression and gene network analyses are limited by the lack of use of a three-group design that differentiates between TE and PTSD. Thus, the first aim of this study was to extend prior expression studies of PTSD, using both TE and non-TE exposed controls. In so doing, we are able to understand what expression signatures are associated with trauma exposure (as seen in differences between TE and non-TE groups) and PTSD (as seen in differences between PTSD and TE groups). The second aim was to provide a system view of the expression signatures using weighted gene co-expression network analysis (WGCNA). While WGCNA identifies sets of genes showing related expression patterns and therefore having likely shared disease functions (34–36), it does not provide information on the biological functions and process the significant modules are enriched for. Thus, all significantly correlated modules were assessed for the enrichment of cellular processes and biological functional categories using gene set enrichment analysis [GSEA; (37)]. These analyses allow us to better understand co-expression networks and potential biological functions that may differentiate those with and without trauma exposure, as well as-among those with trauma exposure-those with and without PTSD. Genome-wide expression data were generated using RNA isolated from Peripheral Blood Mononuclear Cell (PBMC) obtained from an ethnically homogeneous sample of female subjects, known to be at higher genetic risk for PTSD.

## Methods and Materials

### Overview of Larger, Parent Study

Participants were sampled from a larger community sample of young adults. All subjects gave their informed consent before being included in the study. The study was conducted in accordance with the Declaration of Helsinki, and the research protocol was approved by the Institutional Review Board (IRB) at Medical University of South Carolina. These 281 young adults were 59.2% female; primarily Caucasian (84.7% Caucasian, 6.1% African-American, 9.2% Other), and between the ages of 21 and 30 (*M* = 24.76, *SD* = 2.59). Participants were recruited to one of three study groups: non-trauma exposed control (controls), trauma exposed without PTSD (TE), or trauma exposed with PTSD. See prior work for information for eligibility details (38). Of those, 72 controls, 72 TE, and 53 PTSD participants (*n* = 197) provided blood samples for genomic analyses.

### Current Study Sub-sample Participants

For this study, 15 European Ancestry females from each group (*n* = 45) were included. PTSD group participants with the most PTSD symptoms were prioritized for inclusion. PTSD and TE groups were matched on trauma characteristics (i.e., number of traumas, time since traumas) and age. Control group participants were age-matched to the PTSD and TE groups.

There were no differences between groups on age (*F* = 0.27, NS). There were differences in number of experienced traumas between control (*M* = 0.07) and TE/PTSD groups, but not between TE and PTSD groups (*M* = 0.93, *M* = 1.73, respectively; *F* = 11.90, *p* < 0.001). The PTSD group (*M* = 40.79) reported more PTSD symptoms than TE group (19.29; *t* = 15.842, *p* < 0.001). The PTSD group (*M* = 16.53) reported more depressive symptoms than the TE and control groups (*M* = 3.87, *M* = 1.80, respectively; *F* = 954.47, *p* < 0.001).

### Measures

#### Number of Traumatic Events

Using the Life Events Checklist (39), count scores for total endorsed witnessed or experienced traumas was used as a covariate in the differential expression analyses (see Table 1).

**Table 1:** Percent of trauma-exposed (TE) and PTSD groups witnessing or experiencing different types of traumatic events.

	**% TE group**	**% PTSD group**
Natural disaster	42.9	28.6
Fire or explosion	14.3	7.1
Transportation accident	71.4	50.0
Serious accident	21.4	28.6
Exposure to toxic substance	7.1	0.0
Physical assault	35.7	64.3
Assault with a weapon	28.6	35.7
Sexual assault	21.4	50
Other unwanted or uncomfortable sexual experience	14.3	42.9
Combat or war zone exposure	7.1	0.0
Captivity (e.g., being kidnapped)	0.0	0.0
Life-threatening illness	35.7	28.6
Sudden violent death	21.4	14.3
Serious injury you caused to someone else	0.0	14.3
Other stressful event	21.4	21.4

#### PTSD

Using the PTSD Checklist [PCL; (40)], individuals with scores of 30 or higher (41) and who met the minimum symptoms per cluster were given a diagnosis of probable PTSD (Cronbach's Alpha: 0.91). The Mini-International Neuropsychiatric Interview (MINI) PTSD scale was used to confirm PTSD diagnosis (42).

#### Depression Symptoms

a sum of 20 items assessing depressive symptoms using the Beck Depression Inventory [BDI; (43)] was entered as a covariate and additional phenotypic measure in the gene expression (Cronbach's Alpha: 0.92) and network analyses, respectively.

### RNA Isolation

Total RNA containing the small RNA fraction was isolated from 9 ml of whole blood using the mirVana-PARIS kit (Life Technologies, Carlsbad, CA), following manufacturer's protocols. RNA concentration was measured using the Quant-iT Broad Range RNA Assay kit (Life Technologies), and the RNA Integrity Number (RIN) was measured on the Agilent 2100 Bioanalyzer (Agilent Technologies, Inc., Santa Clara, CA). All RNA samples had an excellent RIN scores (average RIN ≥ 9.0, s.d. ± 0.5).

### mRNA Expression Microarrays

Genome-wide expression assays were ran using the Affymetrix (Santa Clara, CA) GeneChip® Human Genome U133A 2.0 (HG-U133A 2.0) following the Affymetrix® protocol (44). This array provides comprehensive coverage of the transcribed human genome using 22,214 probesets, and captures the expression of ≈18,400 human transcripts. Array quality was assessed by estimating the 3′/5′ ratios of GAPDH, and the percentage of “Present” genes (%P) and array exhibiting GAPDH 3′/5′ <3.0 and %*P* > 40% were considered of good quality. Based on these metrics no arrays were excluded.

### Microarray Normalization

Expression values were calculated following the pre-processing procedures: (1) Robust Multiarray Average adjusting for probe sequence (GCRMA) background correction, (2) log_2_ transformation, (3) quantile normalization, and (4) median-polish probeset summarization using **P**artek **G**enomics **S**uite v6.23 (PGS; Partek Inc., St. Louis, MO) (45, 46). The batch effect removal option in PGS was used to control for batch effects. Microarray quality was assessed by principal component analysis (PCA) and unsupervised hierarchical clustering to identify potential outliers. One sample was identified as outlier and removed from future analyses (Supplementary Figure 1).

Microarray reliability was assessed, by validating the expression levels of four genes, (DUS2, FRMD4B, CXCR6, and SRRT) via quantitative real-time PCR using a Taqman approach. We observed a high correlation between the two platforms, i.e., the Pearson correlation ranged from *r* = 0.664 (DUS2) to *r* = 0.846 (SRRT); see Supplementary Table 1 and Supplementary Figure 2.

### Adjusting for Cell Heterogeneity

We used the ImmQuant software (47), which takes as input transcription profiles, and provides the predicted quantities of various cell types within each sample. The deconvolution approach builds on the implementation of the digital cell quantification (DCQ) deconvolution algorithm (48), which is used for immune cell-type prediction in human samples, resulting in gene expression markers for 39 cell types being identified and accounted for to minimize the effect of cell based differential gene expression (Supplementary Figure 3).

### Data Analytic Plan

#### Aim 1: Gene Expression Analyses

Detection of differentially expressed genes between groups was performed in the Number Cruncher Statistical Software (NCSS) v11, using a robust multiple regression model (49), with gene expression as the dependent variable and group as the explanatory variable. To reduce the number of potential covariates and account for potential collinearity between them, the 39 cell-based estimates together with RIN and Batch, were incorporated into a principle component analysis (PCA). The first 6 principle components (PCs) accounted for 13% of the variance in the data and were used as covariates, along with number of interpersonal traumatic events (50) and depressive symptoms (51). Our intention of conducting a PCA here was to reduce the number of covariates, increase our power, and use the higher order orthogonal relationships between the covariates to adjust for their effect on gene expression.

#### Aim 2: Network Analyses

The gene network analyses were conducted using WGCNA. The method for constructing scale-free networks by WGCNA has been described in previous studies (52, 53). The gene co-expression networks were constructed by using the WGCNA v1.36 package in R environment (v3.3.2) using a minimum module size of 35 genes and a minimum module merge height of 0.8. To build the networks, we used all transcripts differentially expressed between the three groups at *p* ≤ 0.05 (i.e., for the PTSD/TE, PTSD/C, and TE/C comparisons a total of 1,701, 441, and 1,846 genes were included, respectively). This significance threshold was chosen to allow for: (**i**) the inclusion of true positive signals with a smaller effect size (which would otherwise be excluded from the more stringent statistical criteria applied in gene expression analyses), (**ii**) retain a sufficient number of genes with biological importance in PTSD and TE for the building of gene co-expression networks, and (**iii**) exclude genes with low variance, i.e., genes with limited importance for the disease trait (49, 54).

Following module definition, the module eigengene (ME) representing the first principle component (PC) of all summarized gene expression profiles within a given module was calculated. The ME was then correlated with the three diagnostic groups, PTSD, TE, and controls, and statistical significance assessed at Bonferroni-adjusted *p* ≤ 0.05 between the groups (i.e., corrected for a number of modules tested). To account for biological or technical confounds, we fitted the regression model of each gene expression value on covariates and then used the residuals from the model as an input for the network analysis.

The rationale for conducting the network analyses within each diagnostic group separately is based on our ability to understand better group differences between subjects with PTSD, TE not reaching the threshold for PTSD, and controls. Our rationale for this approach was further indicated by our desire to test for network preservation between the three phenotypic groups, i.e., identify the precise network modules that differentiate between the PTSD, TE and controls. Thus, with the current analyses, we have increased confidence inferring the gene expression and gene network differences between groups are associated with trauma exposure (TE-C), PTSD (PTSD-TE), or potentially both (PTSD-C). Below are the results of these three comparisons.

#### Pathway Analyses

To identify known biological processes and pathways that our significant modules were enriched for, the gene set enrichment analysis [GSEA; (GSEAv2.0.14)] was used (37, 55). Individual gene lists for each of the gene modules significantly correlated with PTSD and TE status were generated by rank-ordering all differentially expressed genes by their module membership (MM) to each of the trait-associated modules. In GSEA, Affymetrix HG-U133A 2.0 probe IDs were converted to HUGO Gene Nomenclature Committee (HGNC) gene symbols, and in cases of multiple transcripts representing a single gene, the probeset with the highest MM was retained (56). A priori gene sets were obtained from the Molecular Signatures Database v4.0 from the Broad Institute. A total of 1320 gene sets from the Canonical KEGG Pathways subset of the C2: Curated Pathways collection of MSigDB were assessed. Default parameters were then applied to restrict the *a priori* gene set size between 15 and 500 genes, respectively. A total of 186 a priori gene sets were identified, of which in the PTSD/TE comparison, 165 were filtered out due to gene set size parameters (i.e., gene sets <15 and >500 genes) leading to 21 a priori gene sets tested in the final GSEA analysis. In the TE/Control comparison and the PTSD/Control comparison, 16 and 18 a priori gene sets were used (i.e. 170/186 and 168/186 being excluded).

##### Identification of Candidate Hub Genes

Hubs comprise the highly interconnected nodes within the gene network and have been shown to be functionally significant (57). In this study, hub genes were defined by (1) the strength of their intramodular connectivity (i.e., module membership (MM) (58, 59), which measures the strength of the Pearson correlation product moment (*r* ≥ 0.7) between the individual gene expression and module eigengene (ME) and (2) their gene significant (GS) correlation with phenotype. The upper quartile of transcripts with the highest MM and GS (employed for the significant modules) was selected as candidate hub genes.

##### Network Preservation

We used the module preservation statistic Z_summary_, outlined previously (60), to test for module preservation between the three comparisons (i.e., PTSD/TE, PTSD/C, and TE/C). One advantage Z_summary_ statistics has over the more traditional cross-tabulation approaches is that, in addition to overlap in module preservation, it also accounts for the module connectivity pattern and density. The module preservation also requires that a reference network is assigned which then is compared against the test network. The modules from the control groups (in the PTSD/C and TE/C comparisons) were used as reference modules and the modules from TE group were used as reference modules in the PTSD/TE comparisons. We followed previously outlined recommendations for network preservation, i.e., Z_summary_ ≤ 2 implies no evidence for module preservation, 2 ≤ Z_summary_ ≤ 10 implies weak to moderate evidence, and Z_summary_ ≥ 10 implies strong evidence for module preservation. To ensure that module preservation statistics is not confounded by module size (larger module tend to be more preserved than smaller ones, solely due to their size), in addition to Z_summary_ statistics to assess module preservation, we have also used the median rank statistics, that is invariant to module size.

### Power

Power for the differential gene expression analysis was computed using the power analysis statistical software (PASS) version 19 (NCSS, LLC, Kaysville, Utah) assuming an FDR adjusted *p* ≤ 0.0005, corresponding to an FDR of 10% in our analyses. Generally regarded as a rigorous cut-off (61, 62), in our power analysis, the values of the effect size (*R*^2^|C) represents the amount by which the squared correlation coefficient (R^2^) is increased when the test (T) variables are added to the regression model containing the covariates (C). The (*R*^2^|C) was estimated from our gene expression analyses and had an effect sizes ranging between 0.01÷0.06.

### Testing Whether Findings Hold in an Independent Sample

The gene overlap between the civilian and the unique Veteran samples (n=78) was assessed via the hypergeometric test. The test significance was based on the “representation factor (rf)”, i.e., the number of overlapping genes divided by the expected number of overlapping genes drawn from two independent groups, with a value of ≥1 indicating a significant overlap between the gene datasets. The total number of genes was based on all unique 14,800 transcripts on the Affymetrix HG-U133_2.0 array. We limited our examination of effects across samples to all differentially expressed genes between PTSD/TE, TE/C, and PTSD/C comparisons at *p* ≤ 0.005. Our rationale for choosing this *p*-value was based on the compromise between providing a sufficient number of genes for the analysis, while still controlling the Type I error (63).

## Results

### Aim 1: Gene Expression Analyses

*TE vs. PTSD*. At a FDR of 10%, we identified 53 genes with evidence for differential expression between subjects with TE and PTSD (Table 2). Of these, 44 genes were upregulated, and 9 genes downregulated in the PTSD group compared to TE, respectively. The three top significant genes were the growth regulation by estrogen in breast cancer 1 (GREB1) gene (210562_at; *t* = 11.19, *p* = 1.12E-08, *q* = 2.38E-04), the abhydrolase domain containing 6 (ABHD6) gene (221552_at; *t* = 8.41, *p* = 4.61E-07, *q* = 4.91E-03), and the FERM domain containing 4B (FRMD4B) gene (216134_at; *t* = 7.36, *p* = 2.38E-06, *q* = 1.69E-02). Interestingly, compared to *PTSD vs. TE* comparison, we found a much lower number of differentially expressed genes in the *PTSD vs. Control* comparison, with only 5 differentially expressed genes at FDR of 10%. The top gene in this category was represented by the glutamate receptor, ionotropic, N-methyl D-aspartate 1 (GRIN1) gene (205915_x_at; *t* = −6.04, *p* = 2.22E-06, *q* = 1.19E-02) that was under expressed in subjects with PTSD. In the *TE vs. Cont*rol comparison at FDR of 10%, we observed only two genes: the keratin 4, type II (KRT4) gene (214399_s_at; *t* = −6.64, *p* = 4.86E-07, *q* = 0.008178) and the protocadherin gamma subfamily A, 11 (PCDHGA11) gene (211876_x_at; *t* = −6.45, *p* = 7.84E-07, *q* = 0.008178), with both genes under expressed in the TE group. The quantile-quantile (qq) plots of the *p*-value distribution for the gene expression between the three comparisons are shown in Supplementary Figure 4, and the entire list of differentially expressed genes is provided in Supplementary Table 2. The effect sizes for gene expression studies are generally larger than the effect sizes observed in GWAS, which may be one possible reason why the qq plots may appear inflated, without actually being so (64).

**Table 2:** Significant single gene expression analyses between the different comparisons at an FDR of 10%.

**Probeset ID**	**Gene symbol**	***T*-value**	***p*-value**	***q*-value**
**PTSD/TE**				
210562_at	GREB1	11.19	1.12E-08	2.38E-04
221552_at	ABHD6	8.41	4.61E-07	4.91E-03
216134_at	FRMD4B	7.36	2.38E-06	1.69E-02
210049_at	SERPINC1	6.93	4.78E-06	2.54E-02
217144_at	–	−6.22	1.64E-05	4.83E-02
217065_at	–	6.13	1.91E-05	4.83E-02
208389_s_at	SLC1A2	6.13	1.92E-05	4.83E-02
222334_at	C1orf186	−6.12	1.97E-05	4.83E-02
220594_at	OGT	6.1	2.04E-05	4.83E-02
201679_at	SRRT	6.03	2.30E-05	4.90E-02
210782_x_at	GRIN1	−5.74	3.88E-05	6.87E-02
206224_at	CST1	5.63	4.83E-05	6.87E-02
201438_at	COL6A3	5.61	5.01E-05	6.87E-02
216847_at	–	5.6	5.04E-05	6.87E-02
208036_at	OPN1SW	5.52	5.84E-05	6.87E-02
206217_at	EDA	5.51	5.97E-05	6.87E-02
210354_at	IFNG	5.48	6.33E-05	6.87E-02
214382_at	UNC93A	5.48	6.40E-05	6.87E-02
209209_s_at	FERMT2	5.47	6.40E-05	6.87E-02
210431_at	ALPPL2	5.45	6.66E-05	6.87E-02
207506_at	GLRX3	5.43	6.99E-05	6.87E-02
201525_at	APOD	5.4	7.39E-05	6.87E-02
214399_s_at	KRT4	5.37	7.83E-05	6.87E-02
219486_at	DUS2	5.35	8.12E-05	6.87E-02
211469_s_at	CXCR6	−5.33	8.34E-05	6.87E-02
208194_s_at	STAM2	5.31	8.76E-05	6.87E-02
211570_s_at	RAPSN	5.3	8.92E-05	6.87E-02
205364_at	ACOX2	5.28	9.28E-05	6.87E-02
221429_x_at	TEX13A	5.27	9.35E-05	6.87E-02
218651_s_at	LARP6	5.2	0.000108	0.075568
214493_s_at	INADL	−5.18	0.000112	0.075568
208804_s_at	SRSF6	5.17	0.000114	0.075568
222354_at	F11R	5.11	0.000127	0.081852
213475_s_at	ITGAL	−5.09	0.000133	0.08331
218913_s_at	GMIP	−5.06	0.00014	0.08381
214933_at	CACNA1A	−5.05	0.000144	0.08381
205034_at	CCNE2	5.04	0.000146	0.08381
222325_at	–	5.02	0.000153	0.086012
215448_at	–	4.99	0.000161	0.086556
212993_at	NACC2	4.97	0.000167	0.086556
211550_at	EGFR	4.97	0.000167	0.086556
220580_at	BICC1	4.96	0.000171	0.086556
216946_at	HLA-DOA	4.93	0.000183	0.090485
207451_at	NKX2-8	4.9	0.000192	0.09288
218186_at	RAB25	4.86	0.000207	0.09304
205017_s_at	MBNL2	4.86	0.00021	0.09304
207772_s_at	PRMT8	4.85	0.000211	0.09304
215534_at	–	4.85	0.000213	0.09304
207885_at	S100G	4.84	0.000217	0.09304
219759_at	ERAP2	4.83	0.000218	0.09304
219768_at	VTCN1	4.82	0.000226	0.094414
216616_at	–	4.77	0.000249	0.100276
215366_at	SNX13	−4.77	0.00025	0.100276
**TE/C**				
214399_s_at	KRT4	−6.64	4.86E-07	0.008178
211876_x_at	PCDHGA11	−6.45	7.84E-07	0.008178
**PTSD/C**				
205915_x_at	GRIN1	−6.04	2.22E-06	1.19E-02
217144_at	–	−5	3.34E-05	6.93E-02
219838_at	TTC23	−4.69	7.58E-05	8.15E-02
200633_at	UBB	−4.61	9.45E-05	8.85E-02
213695_at	PON3	−4.51	0.000122	0.09248

### Aim 2: Network Analysis

#### PTSD vs. TE Group Comparison

In our first set of network analysis, we identified 14 modules [including the *gray* module (*M*_*grey*_), which contains genes unassigned to any of the other 13 modules (52)] between the PTSD and TE subjects.

**Figure 1:**
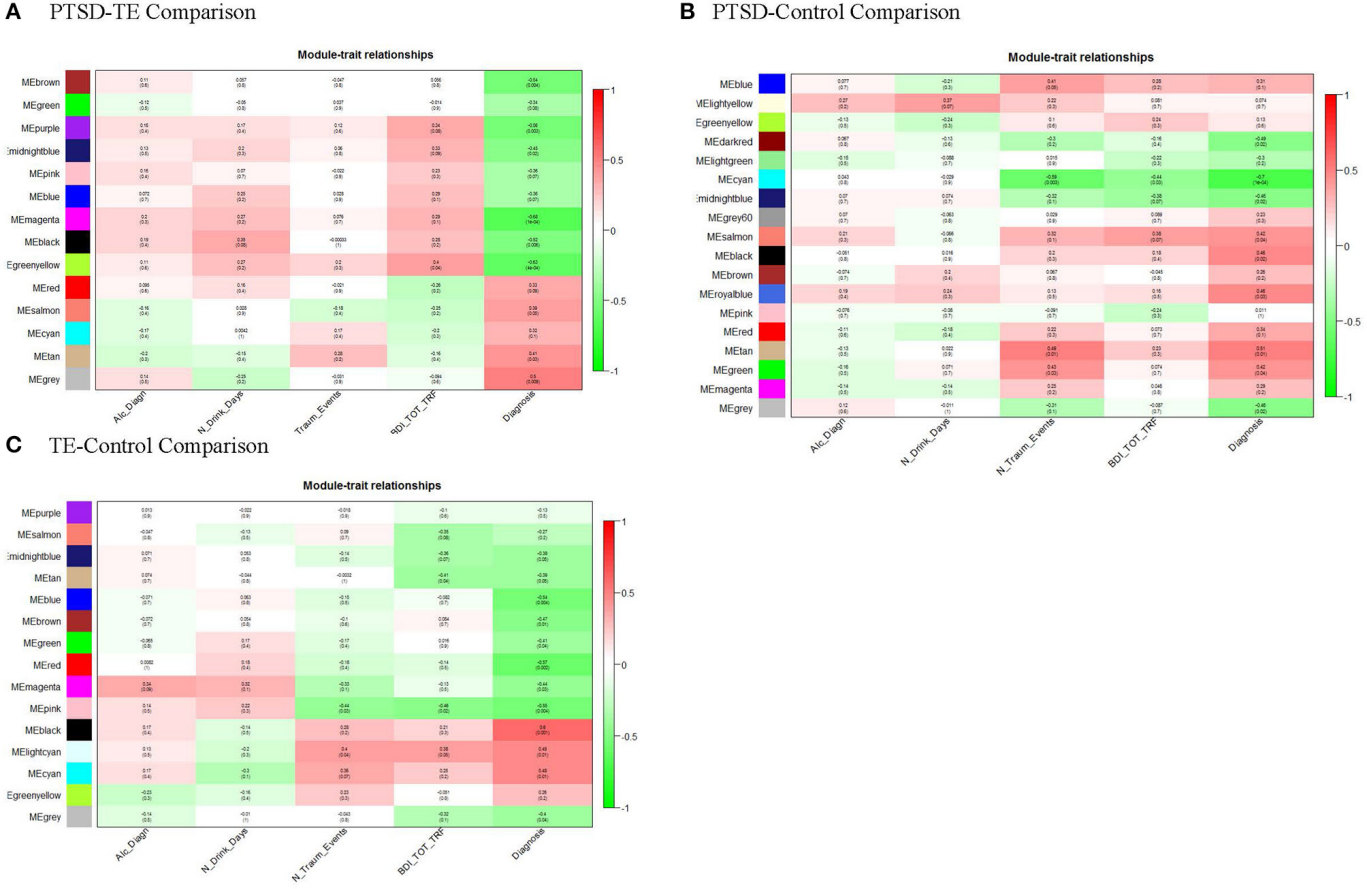
Module-trait relationships. Module MEs are correlated (Pearson) to PTSD/TE case-status (Diagnosis), to identify disease-relevant modules, and to alcohol drinking (Alc_Diagn), number alcohol drinks per day (N_Drink_Days), the number of traumatic events (N_Traum_Events), and Beck depression scale (BDI_TOT_TRF) to assess for confounding of these variables. *P*-values shown are unadjusted for multiple testing. **(A)** After adjusting for number of modules tested, ME_*brown*_, ME_*purple*_, ME_*magenta*_, and ME_*greenyellow*_, are significantly correlated with PTSD/TE status (Diagnosis); **(B)** After adjusting *p*-values for number of modules tested, only ME_*cyan*_, module is significantly correlated with PTSD/Cont status (Diagnosis). **(C)** Similarly, after adjusting *p*-values for number of modules tested, ME_*blue*_, ME_*red*_, ME_*pink*_, and ME_*black*_ modules are significantly correlated with TE/Cont status (Diagnosis).

The size of the 14 modules ranged from 312 transcripts in *M*_*blue*_ to 35 transcripts in *M*_*cyan*_ and *M*_*midnightblue*_. In addition to the main diagnosis phenotype (i.e., the PTSD/TE comparison) the module eigengenes (MEs) of the 14 modules, which represent the sum of gene expression profiles of each module, were correlated to two additional phenotypes: a number of trauma events (N_Traum_Events) and the Beck Depression Inventory scores (BDI_TOT_TRF). Of the 14 modules, the MEs for 4 modules (containing a total of 628 genes) were significantly and negatively correlated with PTSD at an adjusted Bonferroni *p* ≤ 0.004 (Figure 1A).

#### PTSD vs. Control Comparison

In this comparison between the PTSD and Control groups, we identified 18 modules, of which after Bonferroni correction (adjusted *p* = 0.0027) only one module (*M*_*cyan*_) consisting of 52 genes was significantly negatively correlated with the PTSD phenotype (Figure 1B). Interestingly, *M*_*cyan*_ appeared to be also significantly correlated with the number of interpersonal traumatic events as well (adj. *p* ≤ 0.003). Thus, it may be that, to some extent, modules promoting PTSD development are shared with those that differentiate those with varying levels of interpersonal trauma (IPT) exposure.

#### TE vs. Control Comparison

Finally, in this third comparison, we identified 15 modules in total, of which two modules (*M*_*black*_ and *M*_*red*_) remained significant at the Bonferroni adjusted significance threshold (adj. *p* ≤ 0.004) and 2 additional modules (*M*_*pink*_ and *M*_*blue*_) with a suggestive significance (Figure 1C). None of the identified modules appeared to be correlated with depressive symptoms. A table containing correlations and *p*-values of all modules across the three comparisons is provided in Supplementary Table 3.

#### Detection of Network Hub Genes

**Figure 2:**
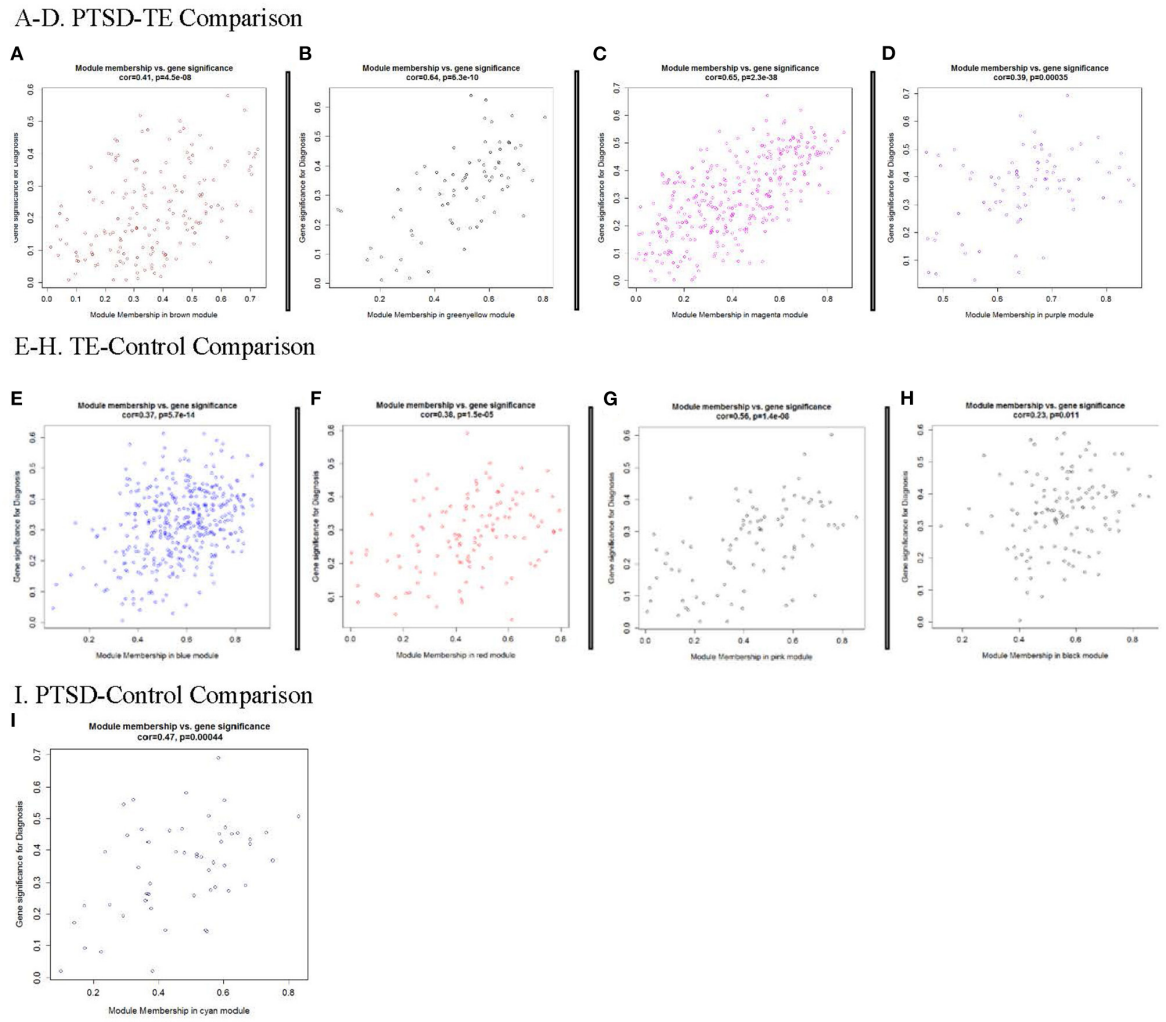
Modules significantly correlated with Diagnosis. Each point represents an individual transcript within each module, which are plotted by the absolute value of their expression correlation to Diagnosis [Gene Significance (GS)] on the y-axis and module eigengene [Module Membership (MM)] on the x-axis. The correlation and corresponding *p*-values are shown for each plot, indicating that increases of GS for transcripts is reflected by the concomitant increase in intramodular connectivity (MM). **(A–D)** Modules significantly correlated with PTSD/TE; **(E-H)** Modules significantly correlated with TE/Cont; and **(I)** Module significantly correlated with PTSD/Cont.

We observed a significant positive correlation between module membership (MM) and gene significance (GS) for all significant modules in the PTSD-TE (Figures 2A–D), in the TE-C (Figures 2E–H), and in the PTSD-C comparisons (Figure 2I), supporting previous observations that genes significantly correlated with the disease are also the most important (or central) elements of the modules (65). Of the 916 genes clustered in the six modules that survive Bonferroni correction, 195 were located in the top quartile of MM and were selected as candidate hub transcripts (see Material and Methods). Interestingly, among these, peptidylprolyl isomerase A (*PPIA*) was shared as a hub between the *M*_*brown*_ and *M*_*pink*_ modules that were significantly correlated with trauma exposure in the *PTSD vs. TE* and *TE vs. Cont*r*ol* comparisons. Full transcript, GS, MM, and gene symbol annotation for candidate hub transcripts are available in Supplementary Table 4.

#### Assessing Module Preservation

**Figure 3:**
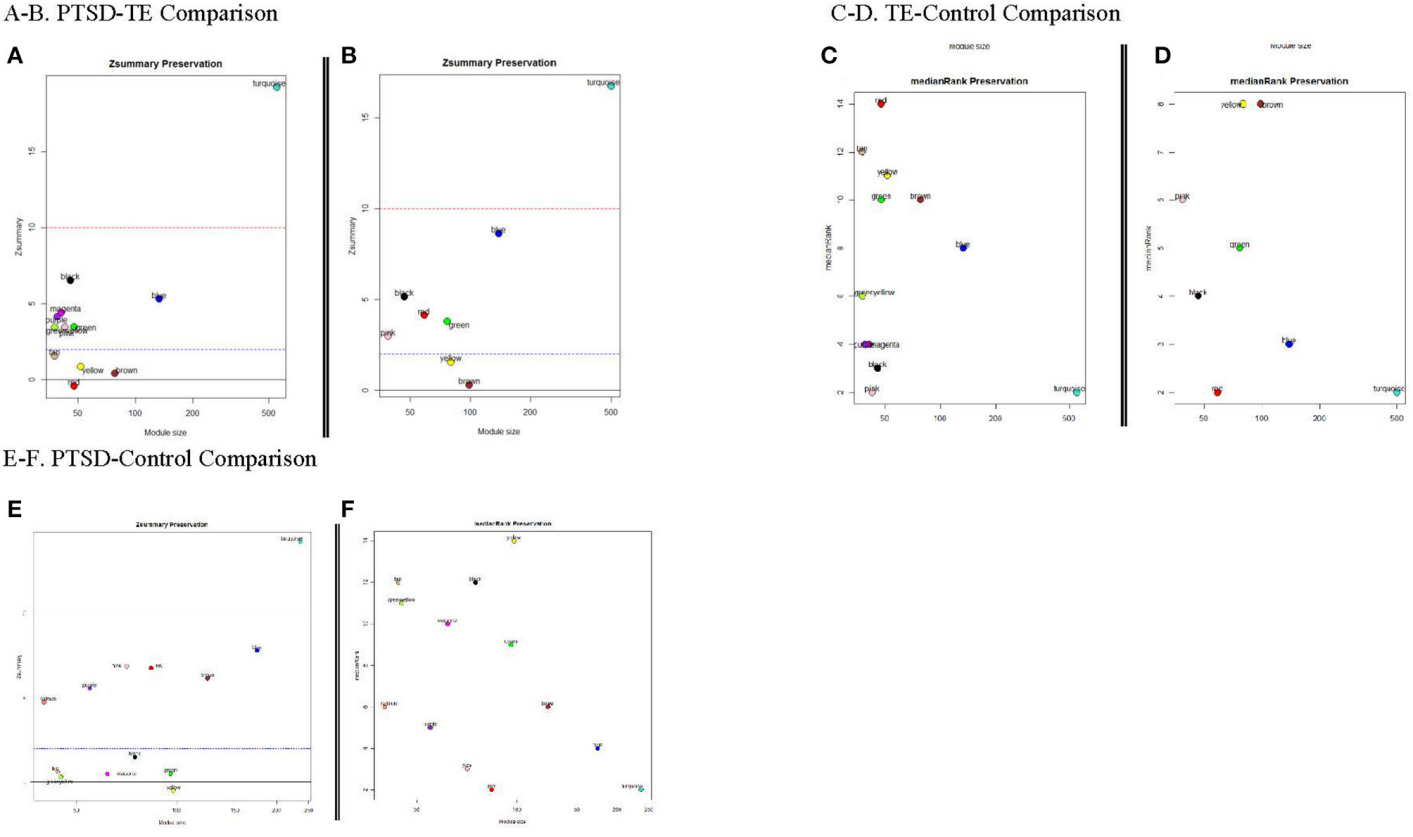
Module Preservation Statistics. Zsummary and medianRank are two mutually complementing preservation statistics to assess module preservation, using either TE or the Cont modules as reference. Y-axis represents preservation statistics for the corresponding module(s) in the case set, and x-axis is the gene numbers in each module. The dashed blue and red lines indicate the thresholds Z ≤ 2 and Z ≤ 10, respectively (see text for more details). **(A,B)** Zsummary and medianRank preservations statistics for PTSD/TE comparison, respectively; **(C,D)** Zsummary and medianRank preservations statistics for TE/Cont comparison; **(E,F)** Zsummary and medianRank preservations statistics for PTSD/Cont comparison.

An important consideration of the network analyses is our ability to test how well-preserved (stable) the modules between the different phenotypes are. To that end, we were interested to know whether any of the modules identified in the PTSD subjects, were also preserved in the Control and/or the TE groups; thus, we used 1,000 permutations in the preservation analysis to answer this question. We observed that except for the large modules (such as the turquoise module ≥1,000 genes) all other modules showed modest to no preservation between PTSD, TE and control subjects (Figures 3A–F) suggesting that the genes in these modules show distinct connectivity and/or density patterns.

#### Pathway Enrichment

Using the default parameters in GSEA (see methods and material), at FDR ≤ 0.2, we identified a total of 16 *a priori* gene sets. We further observed some of these to be shared between more than one module. For example, *a priori* gene sets enriched for the neuroactive ligand receptor interaction, focal adhesion, and vascular smooth muscle contraction gene sets were predominantly shared between *M*_*greenyellow*_, *M*_*magenta*_, and *M*_*brown*_ modules in the *PTSD vs. TE* comparison and in *M*_*blue*_ in the *TE vs. Control*. Similarly, pathways enriched for genes involved in focal adhesion were shared between *M*_*greenyellow*_, *M*_*magenta*_, and *M*_*purple*_ in the *PTSD vs. TE* comparison. In addition to shared gene pathways, we also identified modules with unique enrichments. Among these *M*_*brown*_, identified in the *PTSD vs. TE* comparison, was the only module enriched for genes involved in Alzheimer disease, long-term potentiation and chemokine signaling, while *M*_*greenyellow*_ was uniquely enriched for gene sets in the cytokine receptor interaction pathway. Interestingly, while some of the molecular features of PTSD have been shown to be associated with neuroinflammation, in our study, the only module that was enriched for genes related to immune processes such as antigen processing and presentation, or natural killers cell mediated cytotoxicity pathways was in *M*_*pink*_ from the *TE vs. Control* comparison. In addition to genes involved in neuronal, cellular, and/or neurodegenerative processes we also identified pathways enriched for genes involved in MAPK, calcium, or chemokine signaling. A full list of the significant unique and shared enriched gene sets, as well as descriptions of PTSD biology, is given in Table 3.

**Table 3:** GSEA ontology enrichment results for the significant gene modules.

**PTSD/TE_Brown_Module**	**Size**	**ES**	**NES**	**NOM *p*-value**	**FDR *q*-value**	**FWER *p*-value**	**Rank at max**
KEGG_ALZHEIMERS_DISEASE	16	0.3808411	1.8343772	0.006012024	0.11456045	0.118	564
KEGG_CHEMOKINE_SIGNALING_PATHWAY	28	0.2661027	1.6540613	0.04158416	0.12534362	0.292	685
KEGG_VASCULAR_SMOOTH_MUSCLE_CONTRACTION	16	0.34704605	1.6623145	0.027027028	0.152568	0.283	520
KEGG_LONG_TERM_POTENTIATION	15	0.36446524	1.6956825	0.030592734	0.17543387	0.248	462
**PTSD/TE_GreenYellow_Module**	**Size**	**ES**	**NES**	**NOM** ***p*****-value**	**FDR** ***q*****-value**	**FWER** ***p*****-value**	**Rank at max**
KEGG_NEUROACTIVE_LIGAND_RECEPTOR_INTERACTION	38	0.29289687	2.0918672	0	0.039426543	0.032	244
KEGG_FOCAL_ADHESION	25	0.31623906	1.9317259	0.003802281	0.054069344	0.083	921
KEGG_CYTOKINE_CYTOKINE_RECEPTOR_INTERACTION	28	0.2855701	1.8023701	0.010141988	0.06624937	0.144	602
**PTSD/TE_Magenta_Module**	**Size**	**ES**	**NES**	**NOM** ***p*****-value**	**FDR** ***q*****-value**	**FWER** ***p*****-value**	**Rank at max**
KEGG_FOCAL_ADHESION	25	0.46487573	2.8352916	0	0	0	518
KEGG_VASCULAR_SMOOTH_MUSCLE_CONTRACTION	16	0.3864319	1.9351841	0.004008016	0.04435187	0.067	461
KEGG_NEUROACTIVE_LIGAND_RECEPTOR_INTERACTION	38	0.25406504	1.81941	0.01026694	0.062306367	0.136	381
**PTSD/TE_Purple_Module**	**Size**	**ES**	**NES**	**NOM** ***p*****-value**	**FDR** ***q*****-value**	**FWER** ***p*****-value**	**Rank at max**
KEGG_FOCAL_ADHESION	25	0.36741436	2.2619853	0	0.018515298	0.015	542
**TE/Controls_Blue_Module**	**Size**	**ES**	**NES**	**NOM** ***p*****-value**	**FDR** ***q*****-value**	**FWER** ***p*****-value**	**Rank at max**
KEGG_NEUROACTIVE_LIGAND_RECEPTOR_INTERACTION	41	0.26657942	2.0054896	0.008032128	0.042644728	0.039	679
KEGG_CALCIUM_SIGNALING_PATHWAY	24	0.29648352	1.698969	0.02892562	0.11025008	0.188	601
KEGG_MAPK_SIGNALING_PATHWAY	31	0.23569264	1.5734301	0.052208837	0.13829629	0.316	768
**TE/Controls_Pink_Module**	**Size**	**ES**	**NES**	**NOM** ***p*****-value**	**FDR** ***q*****-value**	**FWER** ***p*****-value**	**Rank at max**
KEGG_ANTIGEN_PROCESSING_AND_PRESENTATION	15	0.49288395	2.3176186	0	0.004643181	0.004	503
KEGG_NATURAL_KILLER_CELL_MEDIATED_CYTOTOXICITY	23	0.32229558	1.7953429	0.016129032	0.080599934	0.137	118

### Results From Independent Sample

We tested whether our initial findings held in an independent sample composed of military Veterans; the data from that sample are described elsewhere (66). Notably, the Veteran sample is primarily male and slightly older than the initial test sample (i.e., 94.5% male, age range 21–40). The extent to which differentially expressed genes were found across samples was assessed via hypergeometric test assaying the level of overlap between the two datasets and represented by a Venn diagram in Supplementary Figure 5 and overlapping genes in Supplementary Table 5. The Venn diagram was drawn through an online source (https://bioinfogp.cnb.csic.es/tools/venny/index.html). Based on this comparison, we observed a significant overlap for the DEG (at unadjusted *p* ≤ 0.005) between our civilian and Veteran samples in the PTSD/TE comparison (i.e., PTSD/TE_Civ (*n* = 283 at least nominally different genes); PTSD/TE_Vet (*n* = 565 at least nominally different genes); gene overlap (n=19); total number of genes (*n* = 14,800; rf = 1.8; *p* = 0.012) only. There was no significant overlap between the other two comparisons (i.e., PTSD/C and TE/C).

### Power Analysis Findings

**Figure 4:**
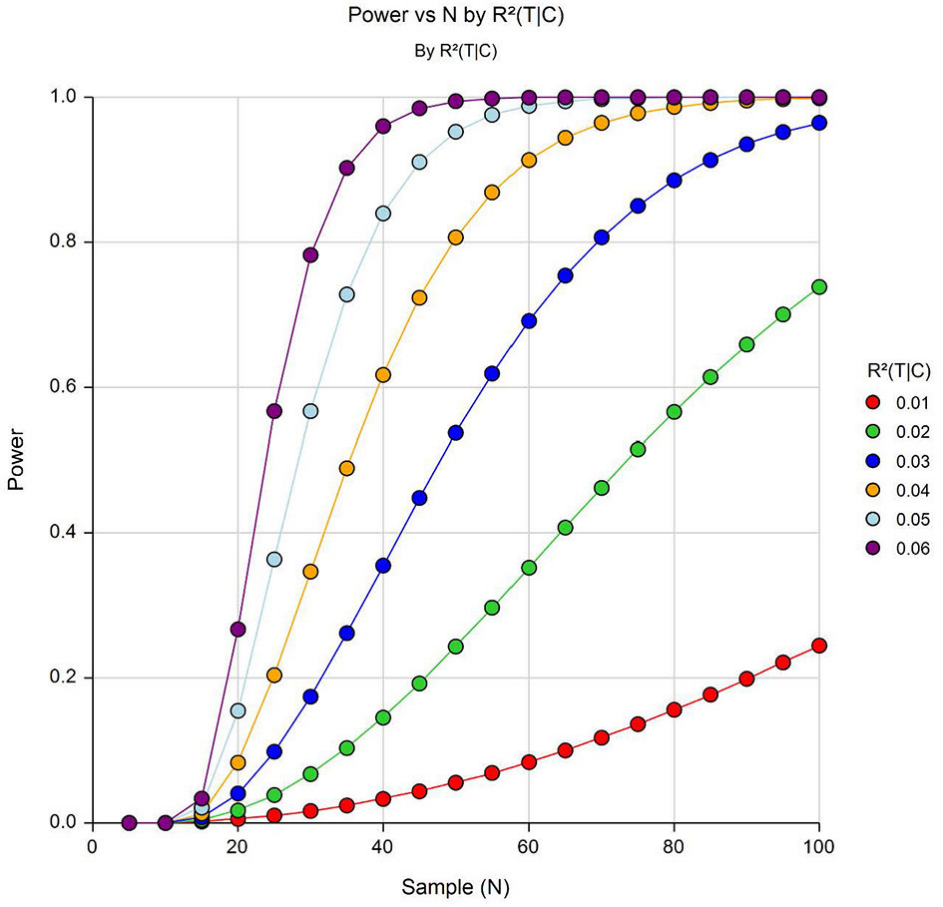
Power to detect differentially expressed genes with varying effect sizes (*R*^2^6C) at α = 5 × 10^−4^. Y-axis shows power and the X-axis shows effective sample size.

As shown in Figure 4, we have a sufficient power to detect an *R*^2^ increase of 6% attributed to one independent variable after adjusting for 9 covariates at an FDR adjusted *p* ≤ 0.0005.

## Discussion

This study employs a three-group design that allowed disaggregation of expression differences among PTSD, TE, and C groups. The largest number of expression differences were observed between PTSD-TE groups (54), followed by PTSD-control (33) and TE-control (2). We observed overlap between those that differentiated the PTSD-TE groups, and PTSD-control groups (hypergeometric *p* = 1.17E-33). Specifically, 19 overlapped across the sets of analyses.

In terms of the network findings, there were 4 modules, 1 module, and 2 modules that differentiated the PTSD-TE, PTSD-C, and TE-C groups, respectively. The only overlapping module was the one that differentiated PTSD-C groups, also differentiating PTSD-TE groups. Supporting previous network findings (32, 33), we identified pathways enriched for genes involved in chemokine signaling and immune system functioning (i.e., natural killer cells; Supplementary Table 4), suggesting these modules captured genes sets involved in the inflammatory response to TE and/or PTSD.

Notably, as none of our significant effects overlapped with genome-wide gene expression findings from prior studies, overlap between our findings and those from related literatures are discussed below. Some of our study findings are consistent with candidate gene literature on PTSD. Specifically, *CNR1*, a cannabinoid receptor gene, was part of a module differentially expressed between PTSD-TE, and prior work found evidence for this gene's association with PTSD (67). The Apolipoprotein E gene *APOE* was also part of a module differentially expressed between these two groups, with research finding evidence for its association with PTSD as well (68). Our findings do not provide support for genes whose SNPs have been implicated in PTSD genome-wide association studies [e.g., *ANKRD55, ZNF626*; (69)].

Some genes differentially expressed between groups were associated with functional processes underlying or related to stress response/PTSD. For example, the nicotinic and muscarinic receptor genes *CHRNA4* and *CHRM2*, respectively, within modules differentially expressed between PTSD-TE, impact immune function and inflammatory responses (70, 71), which are relevant to PTSD. *CHRNA4* also impacts arousal, anxiety, and memory (72), and *CHRM2*, is important in learning, memory and higher order brain functions (73). In addition, the gene *ABHD6*, also differentially expressed between PTSD-TE, and involved in cannabinoid system signaling, is implicated in symptoms of anxiety and depression (74), which are highly likely to co-occur with PTSD (75). The gene *GRIN1*, differentially expressed between PTSD-Controls, encodes a NMDA receptor involved in activation of the pre-frontal cortex and previously linked to Bipolar Disorder (76). Thus, the results from this study suggest that genes differentially expressed between the groups may be functionally involved in immune function and inflammation, stress response, arousal, learning and memory, as well as related psychopathology, including anxiety, depression, and Bipolar.

Recent whole blood, transcriptome-wide mega-analysis comparing those with PTSD to trauma-exposed controls was conducted and notably, while collapsing across trauma types (e.g., interpersonal combat) and sex, no case-control comparisons within the univariate analyses resulted in significant effects withstanding the FDR *p* < 0.05 correction (77). Of the genes differentially expressed between those in the PTSD and TE groups in the current sample, none overlapped with the nominally significant genes differentially expressed in females exposed to IPT in Breen et al. (77). Although no genes overlapped, a number of genes in systems from which genes detected in the current study were found to be (at least nominally) important in differentiating groups. Specifically, the expression of genes involved in innate immune response, the cytokine receptor pathway, and interferon signaling differentiated those with PTSD and TE among females exposed to IPT in both samples.

Of the 283 genes at least nominally differentially expressed between the PTSD and TE groups in the current Civilian sample, 19 of these overlapped with those found to be differentially expressed between the PTSD and TE groups in the Veteran sample. Of these 19 genes that were found to be significant in both samples, some are involved in immune system functioning [e.g., *XIAP*; (78)] signaling processes [e.g., INPP4A; (79)] and learning and motivation [e.g., *EHMT1*; (80)], consistent with prior literature. Others, interestingly, were implicated in tumor growth [e.g., *YES1, PML*; (81, 82)] and a range of other health-related outcomes [e.g., *SNX13* and *CCHCR1*; heart failure, skin disease; (83, 84)]. Thus, over and above trauma type differences between the two samples, it appears that the expression of genes implicated in immune functioning, signal processing, learning/motivation, and broad health-related outcomes differentiate those with trauma exposure with and without PTSD. That is, the expression of genes implicated in these systems arose in two separate samples that differ on target trauma type (interpersonal Civilian vs. combat Veteran), sex (i.e., the civilian sample here was all adult and the Veteran sample was predominantly male), and age [the Veteran age range was higher (21–40) than the civilian (21–30)], which is quite notable. Interestingly, there were no overlapping genes differentially expressed between control and trauma-exposed groups, or between control and PTSD groups across the current study and Veteran samples. As noted above, these two samples differed in terms of the criterion trauma type required, as well as gender and age, so it is perhaps not surprising that two of the comparisons yielded no overlap.

The module preservation statistics further supports our differential expression and network analysis results suggesting separation between the profiles for not only subjects with PTSD and controls, but also for TE individuals. This separation further indicates that different gene modules identified in the PTSD and TE groups are in overall qualitatively different from those in the controls, as well as between each other.

### Limitations, Future Directions, and Conclusions

This study is not without limitations. First, because of its cross-sectional, we are unable to determine the direction of effects. Second, given the stated sample characteristics, these findings may not generalize to males or individuals of other ancestral groups. Although the criterion trauma for the initial sample was interpersonal trauma and for the veteran sample was combat trauma, individuals tended to have other traumas in addition to the target trauma, consistent with prior literature (85). However, additional studies using other trauma types (e.g., disaster, bereavement) as the criterion trauma are needed. It is also important to note that the two samples on which we tested our research questions were quite different—that is, one was primarily female, civilian, and interpersonal trauma-exposed and the other was primarily male, veteran, and combat exposed. Thus, effects found across these two samples really point to gene expression differences that hold over and above sex and trauma type. Future work would benefit from examining these questions across two samples that are more similar on these characteristics. In addition, although we conducted power analyses which suggested we could detect effects explaining an *R*^2^ increase of 6% variance, we, of course, would have missed effects that are smaller than this. Fourth, with any network approaches, our network can capture underlying technical biases rather than actual biological differences, though our investigation of all potential covariates that are present in any genome-wide expression data (i.e., batch effects, RIN, probe dependencies) argues against this possibility. Another limitation is the lack of specific significant thresholds, which we can use to declare whether our PTSD, TE, and control networks are specific to each of these phenotypic traits. We applied permutation-based statistics to account for the preservation significance, i.e., estimating the empirical *p*-value distribution, which is more powerful that the application of predefined *p*-values thresholds. However, it has been shown that this approach is dependent on the module size (86). To alleviate this potential shortcoming, we used an additional preservation measure such as *medianRank* measure that is invariant to the module size. Additionally, as a network is formed by using genes included in the network analysis, we could not examine whether genes outside of this network, i.e., not included in the network analysis (please see Materials and Methods, Network Analysis for our justification) were associated with group membership. Future research should attempt to answer this question. Finally, we chose to use a Microarray because we were interested in examining differentially expressed genes between our three groups, but in the future, RNA-sequencing may have future utility for identification of alternative variants.

Despite its limitations, this study makes several key contributions to the literature. It was an early study to utilize differential expression analyses, including a number of key covariates, as well as network analyses in the examination of expression differences within this three-group design. Broadly these findings suggest that there are more genes and networks differentiating those with and without PTSD, compared to the number that differentiate those with and without TE. This suggests that there is something clinically meaningful about PTSD, that differentiates it from exposure to trauma—in terms of gene expression signatures. Additionally, although there is some overlap, the genes differentially expressed following TE are not the same that are expressed among those with PTSD. These findings are a next step toward identifying potentially novel pharmacological targets for this debilitating condition.

## Data Availability Statement

The datasets presented in this article are not readily available because we as researchers do not have consent from participants to deposit de-identified data in a public repository. Requests to access the datasets should be directed to overall study PI, Carla Kmett Danielson, Ph.D., (danielso@musc.edu).

## Ethics Statement

The studies involving human participants were reviewed and approved by Medical University of South Carolina IRB. The patients/participants provided their written informed consent to participate in this study.

## Author Contributions

KB was PI for this grant, and wrote introduction, part of method, and discussion. VV was Co-PI on this grant and analyzed data and wrote part of method for manuscript. GM and ZT helped with data analysis. GH, DC, CKD and AA were Co-Is on grant and provided critical scientific input on that grant. CKD and AA were also Co-PIs on larger parent grant. AA wrote part of the method section for this manuscript. ZA was Co-I on larger parent grant. All authors provided critical edits to this manuscript.

## Conflict of Interest

The authors declare that the research was conducted in the absence of any commercial or financial relationships that could be construed as a potential conflict of interest.

## Supplementary Material

Supplementary Figure 1 Microarray quality was assessed via a principal components analysis (PCA) on the expression values in which samples were plotted along the first three principal components (PCs) to identify potential microarray outliers. **(A)** Three samples did not load on two of the first three PCs and were removed from subsequent analysis. **(B)** The PCA results were further corroborated via unsupervised hierarchical clustering (using Euclidian metrics), with two of the same samples identified in the PCA analysis also presenting as outliers in the unsupervised hierarchical clustering.

Supplementary Figure 2 Microarray expression data validation using quantitative (qPCR) approach. Expression levels of four genes measured by the expression array-based approach were validated by qPCR in all 45 RNA samples. The Y-axis represents the qPCR data and the X-axis the array data. The correlation coefficients were calculated using the Pearson product-moment.

Supplementary Figure 3 Figure displays the cell deconvolution as a heatmap where each column is a sample, and each row represents a different immune cell type. Each entry (square) in the matrix shows the inferred quantity of a certain cell type in a specific input sample, i.e., the red/blue coloring scheme indicates increase or decrease in estimated cell proportion relative to the control sample.

Supplementary Figure 4 Quantile–quantile (QQ) plots of the gene expression signals across the PTSD/TE, TE/Cont, and PTSD/Cont comparisons. The expected –log10 *p*-values under the null hypothesis are represented on the x-axis, while the observed values are represented on the y-axis.

Supplementary Figure 5 Overlap between civilian and veteran samples.

Supplementary Table 1 Microarray validation using quantitative (qPCR) approach.

Supplementary Table 2A Single gene expression analysis statistics for PTSD/TE comparison. The highlighted rows represent significant DEG at FDR of 10%.

Supplementary Table 2B Single gene expression analysis statistics for TE/Cont comparison. The highlighted rows represent significant DEG at FDR of 10%.

Supplementary Table 2C Single gene expression analysis statistics for PTSD/Cont comparison. The highlighted rows represent significant DEG at FDR of 10%.

Supplementary Table 3A The entire list of genes with their espective gene significance and module membership correlation values across all modules identified in PTSD/Cont comparison.

Supplementary Table 3B The entire list of genes with their espective gene significance and module membership correlation values across all modules identified in PTSD/TE comparison.

Supplementary Table 3C The entire list of genes with their espective gene significance and module membership correlation values across all modules identified in TE/Cont comparison.

Supplementary Table 4 Identified hub genes for the three comparsions. Additional information such as correlation and *p*-values for gene significance (GS) and module membership (MM) are also presented.

Supplementary Table 5 The set of genes overlapping between the civilian and veteran samples at single gene expression level of *p* ≤ 0.005.
