# Supplementary figures and images for: Gene Expression Differences Between Young Adults Based on Trauma History and Post-traumatic Stress Disorder
Source: Front Psychiatry. 2021 Apr 8;12:581093. doi: 10.3389/fpsyt.2021.581093 (PMC8060466; doi:10.3389/fpsyt.2021.581093)

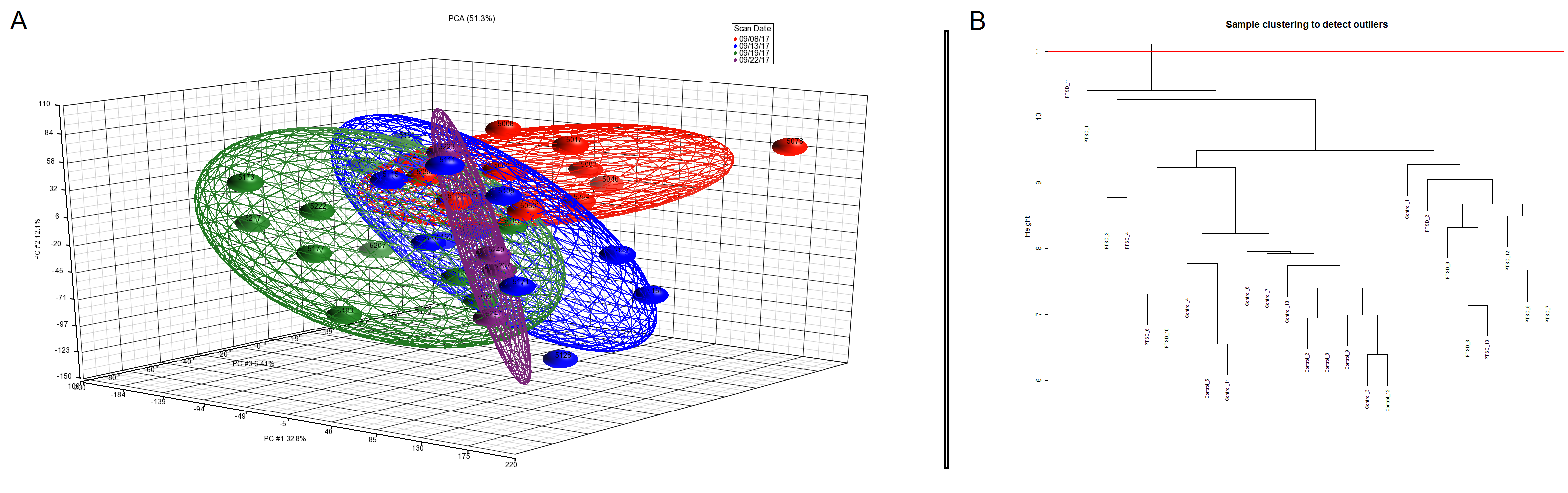

Supplement: Supplementary Figure 1 — Microarray quality was assessed via a principal components analysis (PCA) on the expression values in which samples were plotted along the first three principal components (PCs) to identify potential microarray outliers. (A) Three samples did not load on two of the first three PCs and were removed from subsequent analysis. (B) The PCA results were further corroborated via unsupervised hierarchical clustering (using Euclidian metrics), with two of the same samples identified in the PCA analysis also presenting as outliers in the unsupervised hierarchical clustering. [file Image_1.PNG]

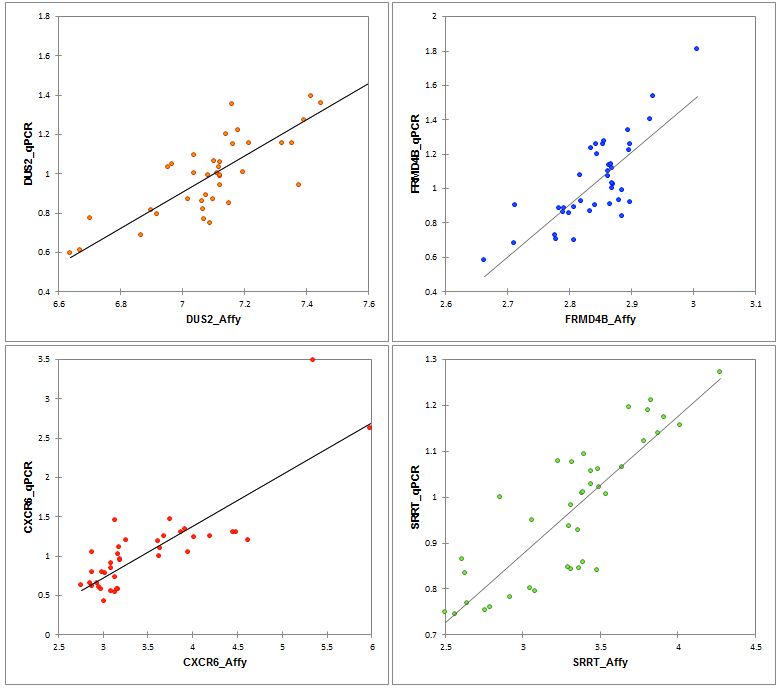

Supplement: Supplementary Figure 2 — Microarray expression data validation using quantitative (qPCR) approach. Expression levels of four genes measured by the expression array-based approach were validated by qPCR in all 45 RNA samples. The Y-axis represents the qPCR data and the X-axis the array data. The correlation coefficients were calculated using the Pearson product-moment. [file Image_2.PNG]

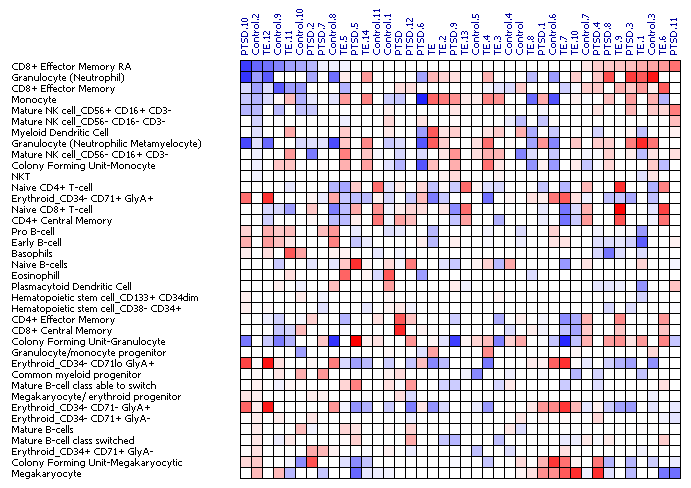

Supplement: Supplementary Figure 3 — Figure displays the cell deconvolution as a heatmap where each column is a sample, and each row represents a different immune cell type. Each entry (square) in the matrix shows the inferred quantity of a certain cell type in a specific input sample, i.e., the red/blue coloring scheme indicates increase or decrease in estimated cell proportion relative to the control sample. [file Image_3.PNG]

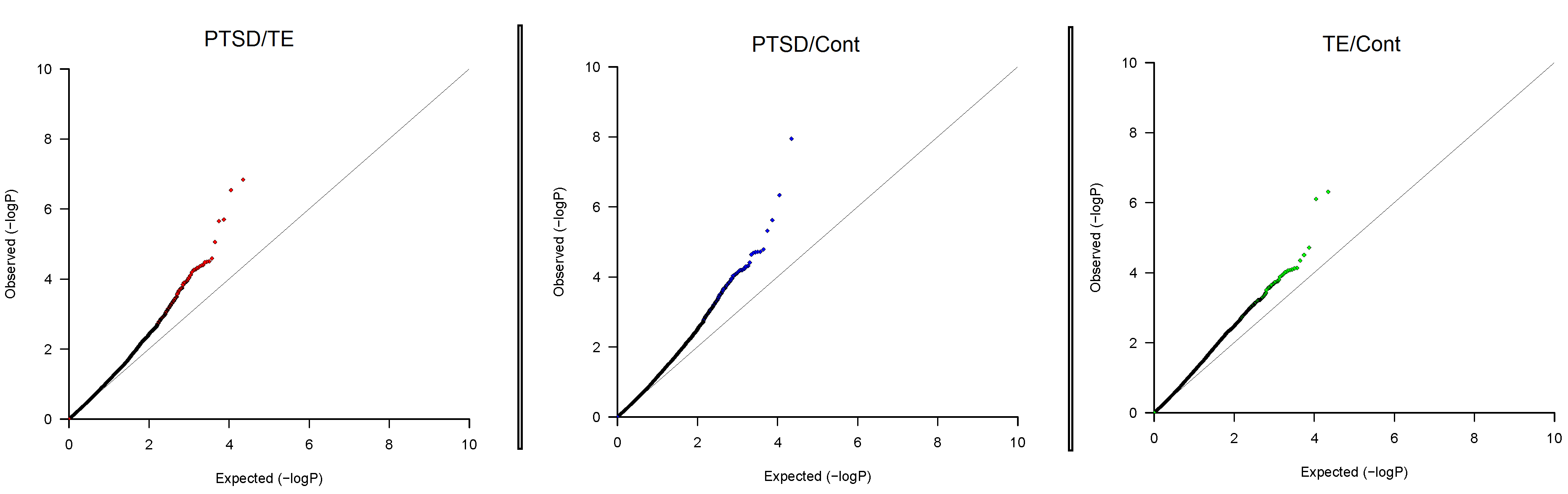

Supplement: Supplementary Figure 4 — Quantile–quantile (QQ) plots of the gene expression signals across the PTSD/TE, TE/Cont, and PTSD/Cont comparisons. The expected –log10 p-values under the null hypothesis are represented on the x-axis, while the observed values are represented on the y-axis. [file Image_4.PNG]

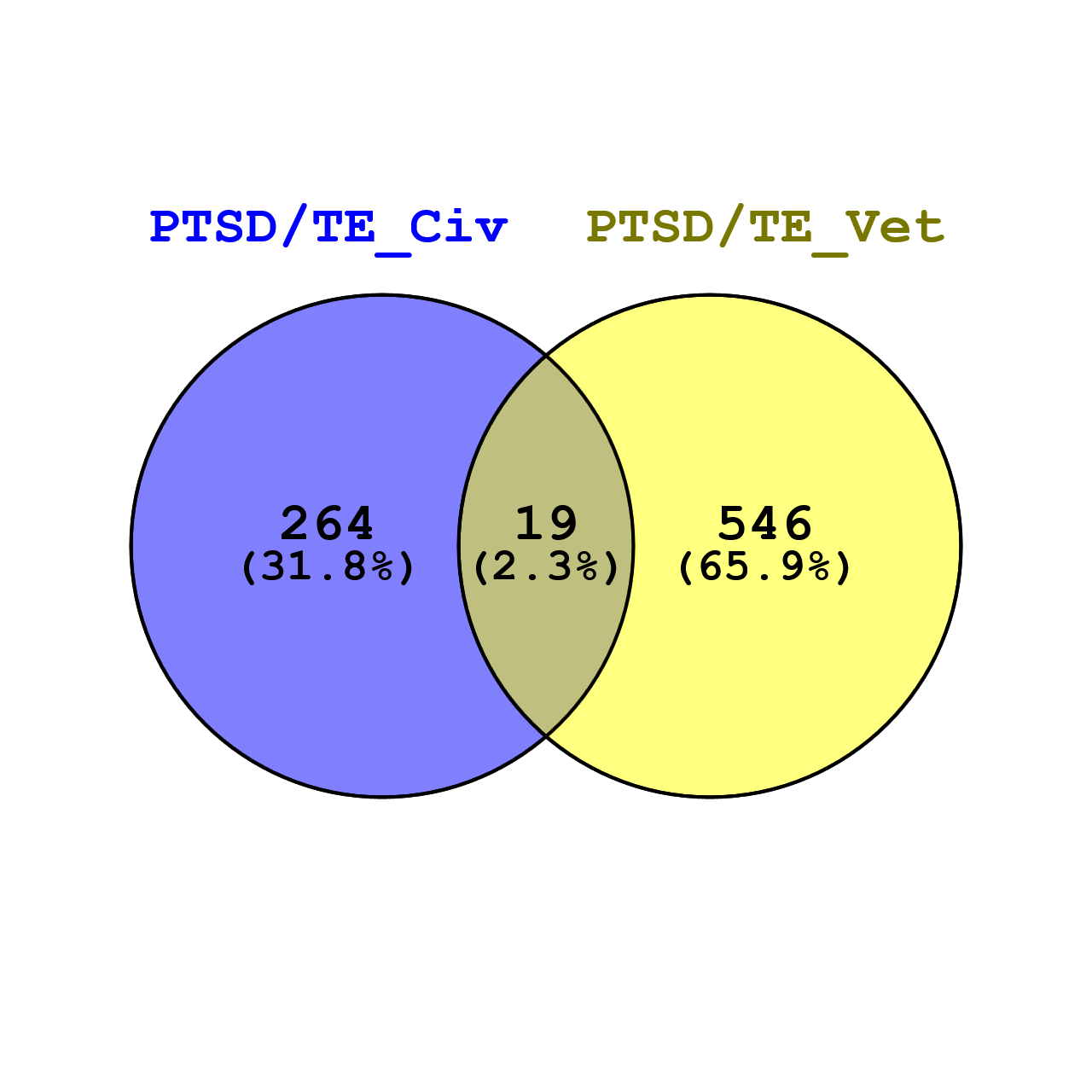

Supplement: Supplementary Figure 5 — Overlap between civilian and veteran samples. [file Image_5.PNG]
